# A statistical calibration tool for methods used to sample outdoor-biting mosquitoes

**DOI:** 10.1186/s13071-022-05403-7

**Published:** 2022-08-17

**Authors:** Halfan S. Ngowo, Alex J. Limwagu, Heather M. Ferguson, Jason Matthiopoulos, Fredros O. Okumu, Luca Nelli

**Affiliations:** 1 Department of Environmental Health & Ecological Sciences, Ifakara Health Institute, Ifakara, Tanzania; 2 Institute of Biodiversity, Animal Health and Comparative Medicine, University of Glasgow, Glasgow, UK; 3 School of Public Health, University of the Witwatersrand, Johannesburg, Republic of South Africa; 4 School of Life Science and Bioengineering, Nelson Mandela African Institution of Science & Technology, Arusha, Tanzania

**Keywords:** Outdoor, Sampling methods, Calibration tool, Mosquitoes, Density dependence, Biting rates

## Abstract

**Background:**

Improved methods for sampling outdoor-biting mosquitoes are urgently needed to improve surveillance of vector-borne diseases. Such tools could potentially replace the human landing catch (HLC), which, despite being the most direct option for measuring human exposures, raises significant ethical and logistical concerns. Several alternatives are under development, but detailed evaluation still requires common frameworks for calibration relative to HLC. The aim of this study was to develop and validate a statistical framework for predicting human-biting rates from different exposure-free alternatives.

**Methods:**

We obtained mosquito abundance data (*Anopheles arabiensis*, *Anopheles funestus* and *Culex* spp.) from a year-long Tanzanian study comparing six outdoor traps [Suna Trap (SUN), BG Sentinel (BGS), M-Trap (MTR), M-Trap + CDC (MTRC), Ifakara Tent Trap-C (ITT-C) and Mosquito Magnet-X Trap (MMX)] and HLC. Generalised linear models were developed within a Bayesian framework to investigate associations between the traps and HLC, taking intra- and inter-specific density dependence into account. The best model was used to create a calibration tool for predicting HLC-equivalents.

**Results:**

For *An. arabiensis*, SUN catches had the strongest correlation with HLC (*R*^2^ = 19.4), followed by BGS (*R*^2^ = 17.2) and MTRC (*R*^2^ = 13.1) catches. The least correlated catch was MMX (*R*^2^ = 2.5). For *An. funestus*, BGS had the strongest correlation with the HLC (*R*^2^ = 53.4), followed by MTRC (*R*^2^ = 37.4) and MTR (*R*^2^ = 37.4). For *Culex* mosquitoes, the traps most highly correlated with the HLC were MTR (*R*^2^ = 45.4) and MTRC (*R*^2^ = 44.2). Density dependence, both between and within species, influenced the performance of only BGS traps. An interactive Shiny App calibration tool was developed for this and similar applications.

**Conclusion:**

We successfully developed a calibration tool to assess the performance of different traps for assessing outdoor-biting risk, and established a valuable framework for estimating human exposures based on the trap catches. The performance of candidate traps varied between mosquito taxa; thus, there was no single optimum. Although all the traps tested underestimated the HLC-derived exposures, it was possible to mathematically define their representativeness of the true biting risk, with or without density dependence. The results of this study emphasise the need to aim for a consistent and representative sampling approach, as opposed to simply seeking traps that catch the most mosquitoes.

**Graphical Abstract:**

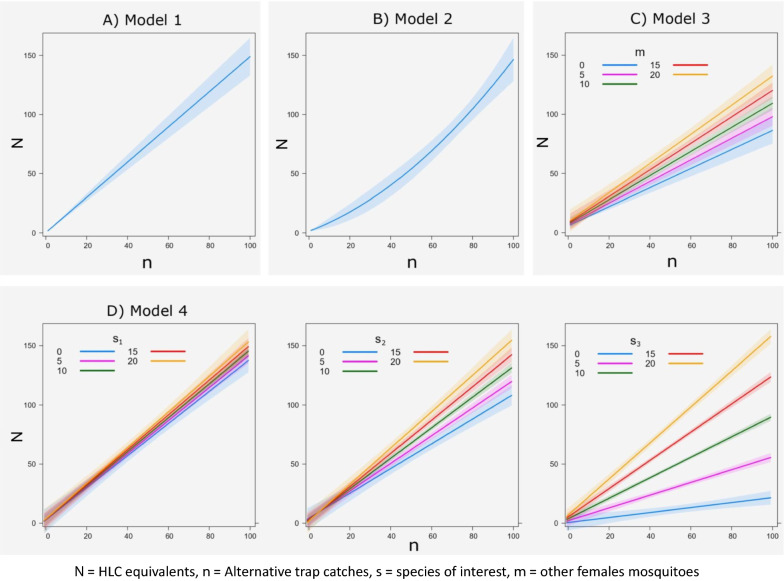

**Supplementary Information:**

The online version contains supplementary material available at 10.1186/s13071-022-05403-7.

## Introduction

Malaria control primarily relies on the use of insecticide-treated nets (ITNs) and indoor residual spraying (IRS) [1–4], with both of these control measures providing protection by targeting mosquitoes that are host seeking or resting indoors. Wide-scale use of these tools has yielded significant progress but challenges remain, such as insecticide resistance [5–8] and outdoor-biting [9–11]. Drivers of outdoor mosquito biting may include human behaviours [12–14], plasticity in mosquito behaviours (e.g. shifting from feeding indoors to feeding outdoors) [7, 15–17] and the effects of some indoor interventions [18, 19]. Sampling mosquito populations is a core component of malaria surveillance activities [20], and the aims of these activities include determining when and where people are most at risk. For the best results, this surveillance should consistently capture the key drivers of biting risk both indoors and outdoors. Unfortunately, representative sampling of mosquito vectors remains a challenge, particularly in outdoor settings.

The main entomological indicators assessed during vector surveillance include human-biting rate (HBR) [16, 21], sporozoite infection prevalence [22, 23], entomological inoculation rate (EIR) [21, 24], time of exposure and proportion of exposure prevented by ITNs [25–28]. The HBR is a fundamental variable for estimating the transmission of malaria and other mosquito-borne diseases [29]. As defined in the Ross MacDonald model, the HBR is required for estimation of the reproductive rate (*R*_0_) of malaria. Both the HBR and sporozoite prevalence are required for calculation of EIR [29], which is calculated as the number of infectious bites a person would be expected to receive in a given location over a given time period. The HBR and EIR are frequently used to estimate the impact of vector control interventions by highlighting how much they reduce exposure [4, 21, 30, 31].

The human landing catch (HLC) has long been the gold standard for direct measurement of human exposure and other key entomological variables. However, this method has several limitations and ethical concerns [32–36] due to its requirement that human volunteers expose parts of their body (usually lower legs) to mosquitoes [26, 37, 38], and this combination of ethical concerns and practical limitations has led to the wide recognition that alternative, exposure-free methods for measuring the HBR are needed [38–42]. Alternatives such as CDC light traps are already widely used for sampling host-seeking mosquitoes indoors [43], but these are unsuitable in outdoor settings. The urgency to identify suitable methods for measuring exposure outdoors is therefore greater [3, 42, 44, 45], especially due to the growing recognition of the importance of outdoor exposure to residual transmission [9, 12, 31].

To date, a number of alternative exposure-free methods have been independently developed and tested in different settings in Africa [3, 25, 38, 40, 42, 45–48]. Some methods provide a good representation of vector species composition and their biting activities, but underestimate density [3, 41, 45]. Others catch more mosquitoes than the HLC and thus overestimate typical human exposure [40, 49]. Finally, there are traps that are easy to implement, but which provide biased estimates of outdoor exposure by disproportionately sampling endophilic rather than exophilic species [50]. These strengths and weaknesses suggest that different traps are optimal for different surveillance applications. Unfortunately, there are no standardised calibration methods to allow estimation of HLC-equivalent exposure from the range of different outdoor sampling methods. Development of a standardised and validated calibration framework for such prediction would enable the results and methods from different studies to be compared. Such a calibration tool would need to reflect the potential non-linear relationship between trap counts and HLC values; this means that no single conversion ‘value’ between methods may apply across the full range of mosquito densities.

Several studies have indicated that trap performance relative to the HLC is density dependent [43, 51], although it should be noted that density dependence is often considered in terms of “intraspecific” density (e.g. the baseline density of the target vector species [42, 51]) but not the density of all mosquitoes, target vectors or not, that are attracted to the trap. However, the mechanisms that could give rise to intraspecific density dependence in trap performance could also generate dependence, with the overall densities of all mosquitoes attracted to the trap, including other species not of interest. While such interspecific dependence on the wider mosquito community is plausible, this has not been formally evaluated in trap evaluation studies.

The overall aim of this study was to provide an extensive comparison of six exposure-free traps for three vectors (*Anopheles arabiensis*, *Anopheles funestus* and *Culex* spp.). Specifically, we aimed to (i) assess the contribution of intra- and interspecific density dependence to trap performance, and (ii) develop an interactive calibration tool (in the form of a Shiny App) through which the number of a given species caught in an HLC can be predicted from catches made by alternative traps.

## Methods

### Study area and vector species

Mosquito trapping data were collected from six adjacent villages in the Ulanga and Kilombero districts of south-eastern Tanzania: Kivukoni (8.2135°S, 36.6879°E), Minepa (8.2710°S, 36.6771°E), Mavimba (8.3124°S, 36.6771°E), Milola (8.3306°S, 36.6727°E), Igumbiro (8.3511°S, 36.6725°E) and Lupiro (8.385°S, 36.670°E). Data were collected over 12 months between 2015 and 2016. The valley has relatively high mosquito abundance which peaks at the end of the rainy season. The common vectors of malaria transmission are *An. arabiensis* and *An. funestus* [16, 24, 52]. Mosquitoes in the *Culex* genera are also highly abundant, with some species being potential vectors for arboviruses found in the study area [53, 54].

### Data collection

Mosquito sampling was carried out during both the wet and dry seasons, using six different traps for sampling outdoor-biting mosquitoes around human dwellings. The traps were: the Mosquito Magnet trap (MMX) [55], BG-Sentinel trap (BGS) [56], Suna trap (SUN) [3], Ifakara Tent Trap-C (ITT-C) [48], M-Trap (MTR) [57], M-Trap fitted with CDC Light trap (MTRC) (this study) and the HLC [3]. Most of these traps have been extensively described elsewhere, with the exception of the MTR fitted with a CDC light trap (MTRC), which was adapted from the original exposure-free M-Trap designed by Mwangungulu et al. [57]. Briefly, the HLC method involved male volunteers aged between 18 and 35 years who sat on a chair with their legs exposed and collected the mosquitoes that attempted to bite, using the mouth aspirator. Mosquitoes were sampled for 45 min each hour, allowing 15 min for rest. Each sampling village had its own set of volunteers.

In the present study, the original MTR was divided into two compartments made of UV-resistant shade netting: one in which a human volunteer sat to attract mosquitoes and the other section in which mosquito are entered [57]. A CDC light trap was suspended inside the other section of the trap to attract more mosquitos to the light source.

The traps were located at least 100 m apart. We assumed that the distance of 100 m offers sufficient independence between the traps as described by previous authors [58, 59]. Initial trap allocation was random, but their positions were switched over successive sampling nights in a Latin square design. In this way each trap was used in each position once over a 7-night cycle. After completion of each cycle, the study team moved to the next village so that one round of sampling in all six villages was completed over 42 trap-nights. Six rounds of data collection were completed, spanning the wettest and the driest periods of the year (252 trap-nights between April 2015 and April 2016). Mosquito sampling was done overnight from 6 pm to 6 am. The collected mosquitoes were morphologically sorted by taxa. A subsample of *An. gambiae* senso lato (s.l.) (*n* = 1405, 26% of total) was analysed by PCR [60] to assess sibling species composition within the complex*.*

### Model fitting

The main goal of our analyses was to create a calibration tool to evaluate outdoor mosquito traps and to validate the tool by comparing the performance of candidate trapping methods relative to HLC (regarded in this study as the “gold standard”). In particular, we wanted to test the shape of the association between the numbers of mosquitoes collected by each trap type with those collected by the HLC. First, we pooled all the hourly collections into a single collection cup per trap per night. Then, for each of the focus mosquito groups (*Culex* genera, *An. arabiensis* and *An. funestus* s.l.), we modelled HLC catches as a function of the catching rate of each alternative trap.

Four general linear models were developed within a Bayesian model fitting framework to allow us to test for linear and non-linear associations through increasing the levels of complexity. The Bayesian approach allowed specific constraints to be placed on the parameters based on biological plausibility; this took the form of priors and uncertainty when converting the counts from alternative traps into HLC-equivalent values in the form of full posteriors.

For any given trap and mosquito group, we defined the response variable $\left( {N_{i} } \right)$ as the number of female mosquitoes on every $i{\text{th}}$ sampling night. Preliminary investigation of the data using Poisson likelihood showed over-dispersion for all three mosquito groups. Our final models did not account for other environmental covariates at specific trap locations (e.g. temperature, humidity). We accounted for the over-dispersion by using a negative binomial likelihood model formulated as a Gamma-Poisson mixture distribution [61]:

$$N_{i} \sim {\text{Poisson}}\left( {{\varvec{\theta}}_{{{\varvec{i}} }} {{\mathbf{\uplambda}}}_{i} } \right)$$

with

$${\varvec{\theta}}_{{\varvec{i}}} \sim {\text{Gamma}}\left( {\tau {{\mathbf{\uplambda}}}_{i} ,\tau {{\mathbf{\uplambda}}}_{i} } \right)$$

where the Poisson rate ${{\mathbf{\uplambda}}}_{{\varvec{i}}}$ is defined by the shape of the relationship between $N_{i}$ and the number of mosquitoes collected with the alternative trap ($n_{i} ,$ Table 1).

**Table 1:** Description of models used to investigate the relationships between female mosquito catches by human landing catch and the alternative traps

Model	Structure
Model 1	$\log \left( {{{\mathbf{\uplambda}}}_{i} } \right) = \beta_{1} n_{i}$
Model 2	$\log \left( {{{\mathbf{\uplambda}}}_{i} } \right) = \beta_{1} n_{i} + \beta_{2} n_{i}^{2}$
Model 3	$\log \left( {{{\mathbf{\uplambda}}}_{i} } \right) = \beta_{1} n_{i} + \beta_{2} n_{i} m_{i}$
Model 4	$\log \left( {{{\mathbf{\uplambda}}}_{i} } \right) = \beta_{1} n_{i} + \sum\limits_{k = 1}^{K} {\beta_{k} {n_{i}} {s_{{k}_{i}}} }$

Since the algebraic form of this relationship is not known, we made three mutually inclusive assumptions with specified mathematical definitions, as follows: (i) that the relationship must start at the origin (i.e. when HLCs catch zero mosquitoes, the other traps will, on average, also collect zero mosquitoes); (ii) that the relationship is positive (i.e. no negative relationships between trap catches); and (iii) that any given trap could potentially suffer from a density effect (i.e. the slope of the relationship is not constant and it can change according to the baseline abundance of mosquitoes, either only of the same mosquito group or of all mosquitoes).

**Fig. 1:**
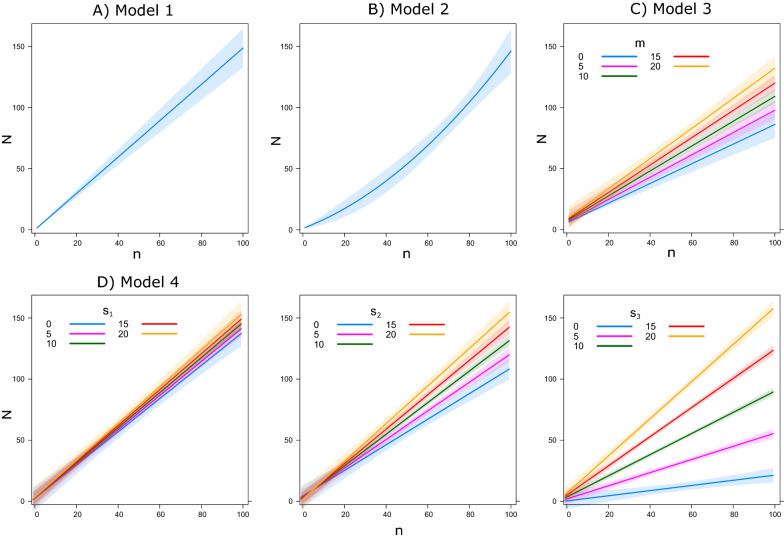
Illustration of models used to investigate the relationship between number of female mosquitoes collected with human landing catch and six alternative traps. *N* = Number of female mosquitoes collected with human landing catch; *n* = number of female mosquitoes collected with a given alternative trap; *m* = pooled female mosquitoes of all the other species collected with the same alternative trap of *n*; *s*_1_, *s*_2_, *s*_3_ = number of female mosquitoes of each of the other* K* species, where *K* refers to all the species collected we developed the focus 1 (here * K* = 3). **A** Model 1 considers a simple linear relationship with* n*. **B** Model 2 considers a quadratic term *n*^2^. **C** Model 3 includes an interaction term between *n* and the number of all females of the other species collected with the same trap (*m*). **D** Model 4 considers all the pairwise interaction between *n* and *s*_1_, *s*_2_, *s*_3_

To define ${{\mathbf{\uplambda}}}_{{\varvec{i}}}$ we therefore formulated four possible scenarios to describe the relationship between HLCs and other trapping methods as summarised in Table 1 and Fig. 1. In Model 1, we considered a simple linear relationship between $N_{i}$ and $n_{i}$ (Table 1; Fig. 1a). In Model 2, we tested if the efficiency of the alternative trap was dependent on the density of the focal mosquito (e.g. “intra-specific” density dependence) by adding a quadratic term $n_{i}^{2}$ (Table 1; Fig. 1b). In Model 3, we tested if the captures of a given group by a given trap were dependent on the abundance of the other taxonomic groups (e.g. “inter-specific” density dependence) by adding, an interaction term between $n_{i}$ and the number of all the females from other mosquito groups collected with the same trap ($m_{i}$) (Table 1; Fig. 1c). Model 4 was similar to Model 3, but we considered all the other $K_{i}$ taxonomic groups separately. Therefore, Model 4 included all the pairwise interaction terms between $n_{i}$ and the number of females of each $k{\text{th}}$ mosquito group $(s_{{k_{i} }} )$ (Table 1; Fig. 1d). Our analysis mainly focussed on three mosquito groups, but we collected a higher number of species hence $K > 3$ (Additional file 1: Table S1).

The analysis was performed in the statistical environment R [62], with Bayesian model fitting to the data done using the program JAGS [63] interfaced within R via the package *rjags* [64]. For parameters $\beta_{1}$, $\beta_{2}$ and $\beta_{k}$ we used a gamma prior (shape = 0.1, rate = 0.1). The prior for $\beta_{1}$ was chosen to ensure a positive relationship between $n_{i}$ and $N_{i}$ and a positive effect of the quadratic and the interaction terms for $\beta_{2}$ and $\beta_{k}$. To achieve convergence, the models were run for up to $3 x 10^{4}$ iterations. Means of posterior distributions with corresponding credible intervals were obtained for each model coefficient $\beta$. We compared different models by their deviance information criteria (DIC) and the goodness of fit of each model using pseudo $R^{2}$ values. Models with the lowest DIC were selected as best. As a further cross-validation, we randomly split the data into a training (75%) and a test (25%) data set, and we calculated the root-mean-square error (RMSE), as the average prediction error by each model.

### Interactive calibration tool

We designed a lookup table (Table 3) containing the means of posterior predictions for different combinations of mosquito taxa, trap types and models. This allowed us to predict the expected number of a given mosquito taxa from an HLC (with credible intervals) based on the number caught in the alternative traps. We also developed an interactive online tool, in the form of an R Shiny App [65] to facilitate these evaluations. This tool provides users with an interactive graphical user interface (GUI) to select the number of captured mosquitoes for a group of interest by trap type, and to explore the predicted number of mosquitoes caught in an HLC by method.

## Results

The statistical correlations between HLCs and other trapping methods for each of the three mosquito groups are summarised in Table 2. The fit of models varied between trap types and mosquito group, with correlations with the HLC (*R*^2^ values) ranging from 0.8 to 53.4% (Table 2). The strength and nature of associations (Models 1–4) varied considerably between mosquito groups and traps; thus, no one single model was best in all cases. We provide an example of a prediction table (Table 3) which describes how mosquito abundance in a HLC can be estimated from catches made by the alternative traps (using Model 1, with intervals grouped by 10). Other model (Models 2–4) outputs/predictions can be easily retrieved from the Shiny App tool. Environmental covariates (temperature and humidity) were dropped during the initial model fitting process as they were not improving the goodness of fit of the model (Models 1–4).

**Table 2:** Summary (*R*^2^, DIC and RMSE values) of models used to investigate the relationship between the numbers of female mosquitoes collected with human landing catch and the six alternative outdoor traps

Mosquito species and model^a^	Alternative outdoor traps^b^											
	SUN				BGS				ITT-C			
	*R*^2^	DIC	ΔDIC	RMSE	*R*^2^	DIC	ΔDIC	RMSE	*R*^2^	DIC	ΔDIC	RMSE
*Culex* spp. (a)												
Model 1	27.3	1026.6	2.36	49.57	25.1	1038.3	0.00	49.37	19.0	769.3	5.07	48.35
Model 2	27.1	1025.3	1.05	49.63	25.1	1045.2	6.88	49.48	19.2	770.2	5.88	48.52
Model 3	27.2	1027.5	3.25	49.66	24.9	1040.6	2.32	49.54	19.4	769.1	4.78	64.60
Model 4	28.1	1024.3	0.00	49.25	25.5	1044.5	6.23	49.15	21.9	764.3	0.00	43.39
*Anopheles arabiensis* (b)												
Model 1	17.4	780.3	0.00	36.69	10.8	549.6	0.61	61.31	9.3	534.7	6.37	64.93
Model 2	17.5	780.3	0.03	36.71	10.8	549.0	0.00	61.35	9.4	528.3	0.00	64.96
Model 3	17.4	782.5	2.21	36.74	10.7	550.9	1.89	61.75	10.4	534.5	6.17	64.16
Model 4	19.4	781.6	1.32	36.06	17.2	551.2	2.19	61.98	11.8	530.6	2.30	64.05
*Anopheles funestus* (c)												
Model 1	14.6	76.5	2.79	8.18	46.6	52.9	0.00	4.24	33.9	111.5	0.00	3.81
Model 2	14.5	77.0	3.30	8.20	50.2	53.7	0.78	4.10	33.6	112.5	0.97	3.83
Model 3	14.5	76.0	2.29	8.20	52.6	53.4	0.50	3.37	34.3	112.4	0.87	3.84
Model 4	16.2	73.7	0.00	8.19	53.4	53.8	0.87	3.96	34.3	112.2	0.69	3.84

Mosquito species and model	MMX				MTR-C				MTR			
	*R*^2^	DIC	ΔDIC	RMSE	*R*^2^	DIC	ΔDIC	RMSE	*R*^2^	DIC	ΔDIC	RMSE
*Culex* spp. (a)												
Model 1	12.4	841.0	12.50	67.48	44.7	1236.5	7.95	42.44	44.2	1174.7	6.00	45.00
Model 2	12.8	838.7	10.27	67.46	44.8	1236.1	7.62	42.38	44.2	1177.1	8.42	45.04
Model 3	12.8	828.5	0.00	67.47	44.7	1228.5	0.00	42.35	44.2	1168.7	0.00	45.09
Model 4	15.9	835.5	7.03	66.15	45.4	1235.8	7.29	44.35	43.8	1172.5	3.77	45.08
*Anopheles arabiensis* (b)												
Model 1	0.8	383.9	3.51	47.19	13.1	991.6	4.43	55.74	11.3	1077.6	12.95	51.96
Model 2	0.4	382.8	2.46	47.30	10.0	1000.8	13.68	55.74	11.3	1073.0	8.37	51.97
Model 3	1.4	385.8	5.42	47.23	13.1	987.1	0.00	55.73	11.3	1070.5	5.84	51.96
Model 4	2.5	380.3	0.00	46.75	12.7	990.5	3.39	55.86	11.1	1064.6	0.00	51.99
*Anopheles funestus* (c)												
Model 1	31.8	40.9	0.62	5.74	32.6	151.1	1.43	5.56	36.8	120.0	0.00	4.62
Model 2	30.6	40.3	0.00	5.79	32.4	149.6	0.00	5.58	36.6	120.6	0.56	4.63
Model 3	30.6	40.7	0.35	5.80	33.4	151.2	1.53	5.51	36.3	122.5	2.45	4.64
Model 4	30.0	40.4	0.09	5.84	37.4	152.8	3.13	5.20	37.4	120.7	0.65	4.59

*DIC* Deviance information criteria, *R*^2^ coefficient of determination, *RMSE* root-mean-square error

^a^See Table 1 for description of models

^b^*SUN* Suna trap, *BGS* BG-Sentinel trap, *ITT-C* Ifakara Tent Trap version C, *MMX* Mosquito Magnet-X trap, *MTR-C* M-Trap combined with CDC light source, *MTR* M-Trap. See “Data collection” section for references pertaining to each trap

**Table 3:** Predicted values for estimating the expected mosquito catches by human landing catch and alternative traps, according to the linear model (Model 1)

Mosquito species	Collected	Expected HLC					
		SUN	BGS	ITT-C	MMX	MTR-C	MTR
*Culex* spp. (a)	10	10 (9–11)	11 (10–12)	8 (8–9)	25 (22–30)	9 (8–9)	9 (9–10)
	20	20 (18–23)	23 (20–25)	16 (14–18)	68 (54–83)	17 (16–19)	19 (17–20)
	30	30 (26–35)	35 (30–39)	23 (20–26)	120 (94–150)	25 (23–28)	27 (25–30)
	40	41 (35–47)	47 (40–54)	30 (26–35)	179 (137–230)	33 (30–37)	36 (33–40)
	50	51 (43–59)	59 (49–68)	37 (32–43)	246 (185–320)	41 (37–46)	45 (40–51)
	60	61 (51–72)	71 (59–83)	44 (37–51)	318 (236–418)	49 (44–55)	54 (48–61)
	70	71 (59–84)	83 (69–98)	51 (43–59)	395 (290–525)	56 (50–63)	63 (55–71)
	80	81 (68–97)	96 (79–114)	57 (48–68)	476 (347–639)	64 (57–72)	72 (63–81)
	90	92 (76–110)	108 (89–129)	64 (53–76)	562 (406–760)	72 (63–81)	80 (70–91)
	100	102 (84–123)	121 (99–145)	70 (59–84)	652 (467–888)	79 (70–90)	89 (77–102)
*An. arabiensis* (b)	10	14 (12–16)	20 (16–25)	20 (17–24)	18 (14–22)	13 (11–14)	16 (14–18)
	20	31 (25–37)	51 (38–66)	50 (39–64)	43 (30–57)	27 (23–32)	36 (31–43)
	30	49 (39–60)	87 (62–117)	86 (63–112)	71 (48–99)	43 (36–51)	59 (49–71)
	40	68 (53–85)	126 (88–175)	125 (90–166)	102 (66–146)	59 (48–70)	84 (68–102)
	50	88 (67–111)	170 (115–239)	168 (118–227)	135 (85–198)	75 (61–91)	109 (88–135)
	60	108 (82–139)	216 (144–308)	213 (147–292)	170 (105–253)	92 (74–112)	136 (108–170)
	70	129 (97–167)	264 (174–382)	261 (178–361)	207 (125–311)	109 (87–134)	164 (129–207)
	80	150 (112–196)	315 (204–460)	311 (209–435)	245 (145–373)	127 (100–157)	192 (150–244)
	90	172 (127–226)	368 (235–543)	363 (241–512)	284 (166–437)	144 (114–179)	222 (172–283)
	100	194 (142–257)	423 (268–629)	417 (275–592)	325 (187–504)	162 (127–203)	251 (194–323)
*An. funestus* (c)	10	2 (1–7)	22 (3–67)	7 (3–13)	5 (1–16)	10 (5–16)	7 (3–11)
	20	3 (1–12)	63 (4–237)	12 (3–28)	9 (1–35)	19 (8–37)	12 (5–23)
	30	3 (1–17)	118 (4–497)	17 (4–44)	13 (1–58)	29 (10–60)	17 (6–36)
	40	4 (1–21)	185 (5–840)	22 (4–60)	17 (1–81)	39 (12–85)	22 (6–48)
	50	4 (1–26)	263 (5–1262)	27 (5–77)	21 (1–106)	49 (14–111)	26 (7–61)
	60	5 (1–30)	352 (6–1760)	32 (5–95)	26 (1–131)	59 (16–139)	31 (8–74)
	70	5 (1–34)	452 (6–2332)	37 (6–113)	30 (1–158)	69 (18–167)	35 (9–86)
	80	6 (1–38)	561 (6–2975)	42 (6–131)	34 (1–185)	79 (20–196)	40 (9–99)
	90	6 (1–42)	679 (7–3689)	47 (6–149)	38 (1–213)	89 (21–226)	44 (10–113)
	100	7 (1–46)	807 (7–4471)	51 (6–167)	43 (1–242)	99 (23–256)	49 (10–126)

Numbers in the second column refer to the number of mosquitoes collected with a given trap. To obtain the estimate of the equivalent number that would be collected with human landing catch (HLC), refer to the column corresponding to the trap itself. Numbers in brackets are (95% credible intervals)

### *Anopheles arabiensis*

**Fig. 2:**
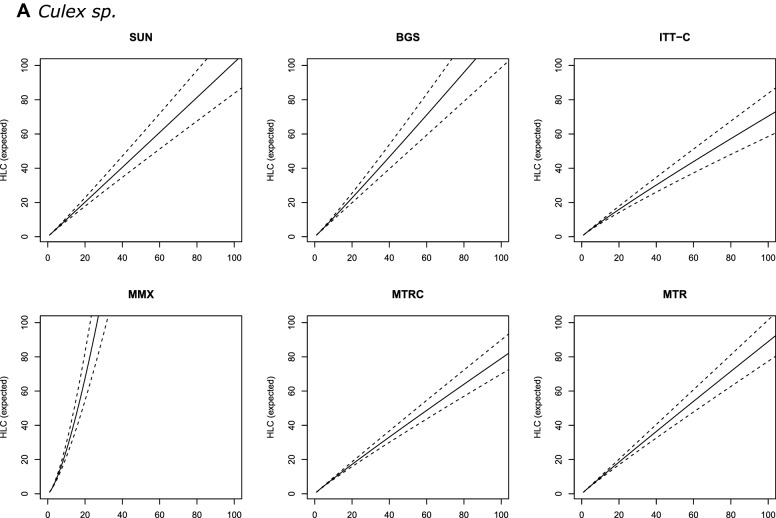

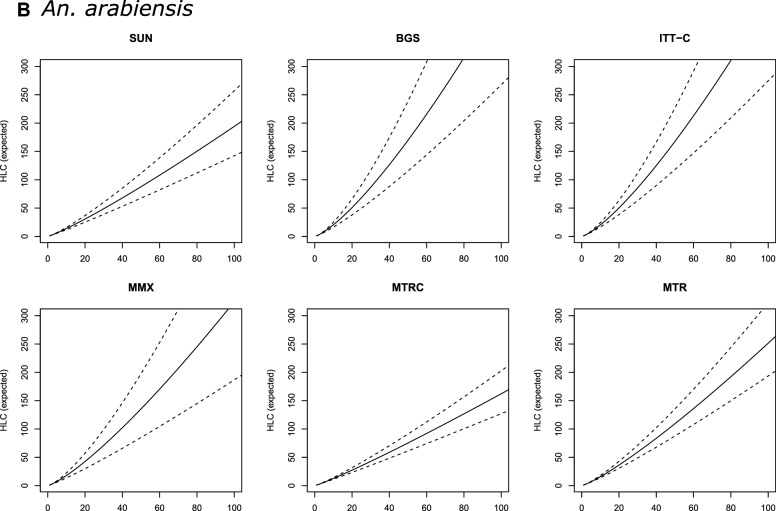

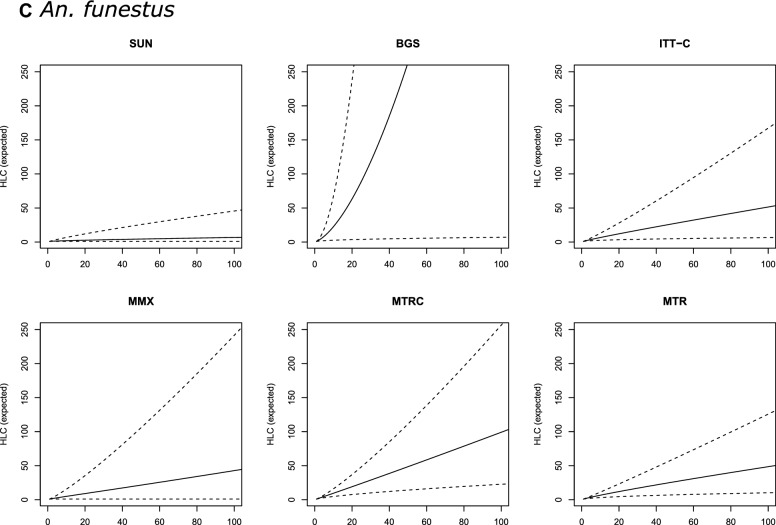
Expected number of female *Culex* spp. (**A**) *Anopheles arabiensis* (**B**) and *Anopheles funestus* (**C**) mosquitoes collected with HLC (*y*-axis), given the number of females collected with alternative traps (*x*-axis). Continuous line is the prediction of a Gamma-Poisson model assuming a linear relationship; dashed lines are 95% credible intervals. Abbreviations: HLC, Human landing catch; SUN, Suna trap; BGS, BG-Sentinel trap; ITT-C, Ifakara Tent Trap version C; MMX, Mosquito Magnet trap; MTRC, M-Trap-Trap combined with CDC light source; MTR, M-trapTrap

In most of the models, trap catches of *An. arabiensis* were only weakly correlated with HLC counts [Table 2 (b)]. SUN was the only alternative trap with a consistent correlation with the HLC of ≥ 17% (*R*^2^ > 17). For this trap, the relationship with HLC catches was best described by Model 4 [*R*^2^ = 19.4; Table 2 (b)], which incorporates both intra- and interspecific density dependence. However, the DIC values however did not vary much between models of differing complexity [ΔDIC = 1.32; Table 2 (b)]. Overall, SUN consistently underestimated HLC catches (for example 100 mosquitoes collected with SUN corresponded to 194 HLCs [95% credible intervals (CIs): 142–257; Table 3 (b); Fig. 2b).

BGS was the only trap where *R*^2^ values substantially increased with model complexity. Here, the most complex model (Model 4), which incorporated intra- and interspecific density dependence, had an *R*^2^ value of 17.2% [Table 2 (b)]. For all other trap types, correlations with the HLC were best explained by the simplest linear relationship (Model 1). Collections from BGS also underestimated the number of mosquitoes caught by HLC, and to a larger extent than the SUN [e.g. 100 mosquitoes caught by BGS is equivalent to 423 (95% CIs: 268–629) by HLC; Table 3 (b); Fig. 2b].

The MMX trap had the poorest correlation and was least representative of the HLC (*R*^2^ range: 0.8–2.5%), particularly at low densities where it often failed to capture any individuals. This trap therefore also significantly underestimated the catches relative to HLC [for example 100 catches of MMX is equivalent to 325 (95% CI: 187–504); Table 3 (b); Fig. 2b].

### *Anopheles funestus*

There were no major differences between the alternative models when describing associations between HLC and the other traps for collecting *An. funestus*. Thus, on the basis of parsimony, we concluded that the simple linear model (Model 1) was sufficient to describe these relationships. BGS was the most highly correlated with the HLC (*R*^2^ range: 46.6–53.4%). The highest *R*^2^ value was from the most complex model (Model 4). However, similar to *An. arabiensis*, the BGS underestimated the number of *An. funestus* caught by HLC [Table 3 (c)] while, in contrast, the MTR, MTRC, ITT-C and MMX traps were only moderately correlated with HLC (*R*^2^ range: 30.0–37.4%); the SUN trap was the worst performing trap for this species [Table 2 (c)].

In general, predictions obtained with all *An. funestus* trap models (Models 1–4, for all trap types) were characterised by very large credible intervals (Fig. 2c), meaning that there was insufficient precision to define a useful calibration factor. This large uncertainty amount of HLC-equivalents of trap catches was particularly pronounced at higher *An. funestus* densities. In that sense, the trap that resulted in a (relatively) narrower prediction was MTR, where for 100 mosquitoes collected, the model would estimate 49 HLC-equivalents, with 95% CIs ranging from 10 to 126 [Table 3 (c)].

### *Culex* species

Overall, there were moderate correlations between the alternative traps and HLC for *Culex* catches compared to those for the *Anopheles* groups (Table 2). However, there were no major differences between the tested models (based on ΔDIC estimates); thus, the simplest linear model was adopted based simply on being the thriftiest. Full details of all models are presented in Table 2 (a). Catches from MTRC and MTR traps had the highest correlations with HLCs (*R*^2^ range: 44.2–45.4%). On the other hand, MMX and ITT-C were the worst performing traps and significantly underestimated the HLC catches [Table 2 (a)].

### Interactive calibration tool

To support detailed assessment and comparison of these and any future trap types for outdoor-sampling, we developed an interactive calibration tool incorporating the key parameters as identified in the analysis above. This tool is designed with simple user interfaces to simplify model inputs and outputs. For example, reporting full conversion tables for Models 3 and 4, which include density dependence, would be challenging since the associated interaction terms would require every possible combination of mosquito group, trap type and catch range. To obtain estimates according to these models, readers can use of our interactive online tool, which is available as an R Shiny App. The coefficients of these models will be updated regularly as additional data are gathered. This tool may be expanded to cover additional geographic regions and mosquito species not currently captured. The tool is hosted by an online server of the “Boyd Orr Centre for Population and Ecosystem Health” (University of Glasgow), and it is freely available at https://boydorr.gla.ac.uk/lucanelli/trapcalibration/.

## Discussion

Despite the growing importance of outdoor-biting mosquitoes and their role in malaria transmission in different settings, there are limited methods for sampling outdoors. HLCs remain common and are sometimes considered to be the gold standard, but there are multiple ethical, cost and logistical concerns limiting its application [66, 67]. Multiple alternative tools have therefore been tested as potential HLC replacements in different settings [25, 39, 41, 43, 48, 50, 68]. While most efforts have focused on finding an alternative that catches as many mosquitoes as the HLC, it is now recognised that what matters more is how representative the catches from any specific trap are relative to HLCs. This means that efforts to improve surveillance methods should include not just new traps, but also a statistical tool for assessing their representativeness.

In this study, we therefore developed and validated a statistical framework for predicting credible intervals of HLC-derived exposure rates based on catches from multiple exposure-free alternatives. We have provided extensive comparison and correction factors for the different trapping methods, as well as evidence for the most representative alternative to the HLC. Furthermore, we have translated the results of our modelling approach into an easy-to-use interactive calibration tool that generates the expected means and credible intervals of nightly HBRs (using HLC as a proxy) based on inputs of other trap catches.

Among the several trapping methods that have been proposed for outdoor mosquito sampling of malaria vectors, only a few have been calibrated relative to the HLC [43], and even fewer have been calibrated in the outdoor setting [42, 45]. These traps provide disparate levels of efficacy relative to the HLC, and they rely primarily on two mutually inclusive principles: (i) the substitution of human subjects with human odours and a carbon dioxide source [4, 46]; or (ii) a trap design that protects human volunteers from bites with physical barriers [25, 41, 45, 57]. Many studies have assessed the correlations between mosquito abundance as estimated from the HLC and an alternative trap [3, 38, 40, 41, 45, 48, 51, 69, 70], but only a few provide the relevant quantitative estimates of “accuracy” (i.e. how close the estimates are to the HLC) and precision (i.e. how variable the estimates are) [38, 40, 41, 45, 48]. Furthermore, to our knowledge, none have provided an explicit calibration tool to facilitate rapid predictions of mosquito counts from an alternative trap into an HLC-equivalent. Such a calibration tool would need to reflect the potential non-linear relationship between trap counts and HLC values, which means that no single conversion “value” between methods may apply across the full range of mosquito densities. This hypothesis is backed up by a multi-country study which evaluated the limitation of CDC light traps on African malaria vectors after observing the non-linearity [43].

In general, the overall measure for goodness of fit (*R*^2^) for models predicting HLC counts was highest in *An. funestus*, followed by *Culex* spp. and *An. arabiensis*. Despite the higher value of *R*^2^ in *An. funestus*, the wider credible intervals were probably due to the much small sample size of this species (total mosquito caught with HCL: *An. funestus* = 226, *An. arabiensis* = 5282, *Culex* = 7191), although it could also have been affected by other ecological features that were not directly captured with this study (e.g. other environmental conditions apart from humidity and temperature). During the model fitting exercise, temperature and humidity were excluded via the model selection process. The proportion of *An. funestus* in the study area compared to other species such as *An. arabiensis* and *Culex* has been historically low [10, 16, 24] although the former species carries a significant amount of infection compared to other commonly known malaria vectors [24].

The performance of some alternative traps in comparison to the HLC has been shown to be density dependent in several investigations [43, 51] although such density-dependent impacts are usually only considered in terms of “intraspecific” dependencies, such as the baseline density of the target vector species [42, 51], overlooking the larger mosquito community. However, the same mechanisms that cause intraspecific density dependence in trap performance may also cause dependence on the overall densities of all mosquito species lured to the trap, including species that are not of public health importance. While such reliance on the wider mosquito community is plausible, it has yet to be tested in trap evaluation studies. Therefore, the present study and the calibration tool that we developed also included a robust assessment of how density dependence may play a role. Models 3 and 4 included these variables and will allow users to incorporate these as covariates when predicting outdoor-biting rates in their settings of interest.

Overall, this study found little evidence that the relative performance of the trapping methods investigated here is modified by the density of the target mosquito taxa or other members of the mosquito community. Models that incorporate intra- or interspecific density dependence in trap performance did not yield any substantial improvements over those assuming simple linear relationship between mosquito counts in the HLC and the alternative method. This indicates that neither intra- nor interspecific density dependence has a large impact on the relative efficiency of the alternative traps tested here. Given the wide range of trap catches, the calibration tool we developed here allows users to incorporate such density-dependence effects (both within and between species) and to examine if these are applicable in their settings. Previous studies detected (intraspecific) density dependence in the performance of some trapping methods [45, 48, 49], but evidence of density dependence in trap performance can be variable even for the same trapping method. For example, studies investigating the performance of the Mosquito Electrocuting Trap relative to the HLC have detected density dependence in some cases [25, 43], but not others [45].

One limitation of this study is that while the HLC is broadly considered to be the gold standard for collecting host-seeking mosquitoes both indoors and outdoors, we only focussed on traps for outdoor sampling. Although we compared a large number of trap types commonly used in Africa settings, other traps may perform differently and potentially better than some of the candidate traps investigated here [25, 41, 45]. Additional studies including additional alternative traps for indoor and outdoor use would be of further value—with the calibration tool developed here providing a useful framework for their evaluation and comparison. Also based on the results presented here, we recommend that for whatever trap used, the users should generate credible estimates of what the HBRs (as estimated from HLC) could be. Due to the potential variation in trap performance between different ecological settings and mosquito species, we do not yet recommend any one specific trap as the best replacement for the HLC. Instead, we recommend that users consider and define the statistical relationships between a prospective trap and the HLC when planning surveillance and interpreting results. The interactive conversion tool we have developed here can be used for that purpose and is now available online as a Shiny App interface.

## Conclusion

Methods for sampling outdoor-biting mosquitoes are urgently needed to improve surveillance of vector-borne diseases. Even if an alternative traps do not catch as many mosquitoes as HLC, it is desirable to define the statistical relationship between them so that credible ranges of actual biting risk can be predicted in units of HLC equivalents. In this study, we successfully evaluated six different outdoor traps and developed a calibration tool to assess their performance relative to the HLC. This tool was validated using data from year-round field collections and enabled a framework for predicting HLC-derived exposure rates representative of individual risk to mosquito biting. The tool incorporates multiple models, including two that allow assessment of effects of both inter- and intra-specific density dependence of the performance of candidate traps. In the specific field trials from which data were obtained here, density dependence between and within mosquito species influenced the performance of only one trap, the BGS, but not any others. An interactive Shiny App calibration tool was developed for this and similar applications. We conclude that this calibration approach provides a valuable framework for assessing human exposure from different outdoor trapping methods. As the performance of candidate traps relative to the HLC varied between mosquito taxa, there was no single optimum. While all the candidate traps underestimated HLC catches, and thus HBRs, the calibration tool created here enables a mathematical definition of the traps relationship as well as model-fitting limits. Further studies of trapping methods and associated evaluation criteria should focus on consistency and representativeness as opposed to simply finding traps that catch as many mosquitoes as HLC.

## Supplementary Information

**Additional file 1: Table S1.** Summary of all other mosquitoes collected for each trap type.

## Data Availability

The R-codes used for analysis and the trap data are available upon reasonable request to the author. The *Shiny App* is accessible by the link provided in the manuscript.

## Abbreviations

DIC: Deviance information criteria
BGS: BG-Sentinel
CDC Light Trap: Centre for Diseases and Control light trap
CIs: Confidence Intervals
DIC: Deviance Information Criteria
EIR: Entomological inoculation rate
GUI: Graphical User Interface
HBR: Human Biting Rate
HLC: Human Landing catches
IRS: Indoor Residual Spray
ITNs: Insecticide Treated Nets
ITT-C: Ifakara Tent Trap type C
JAGS: Just Another Gibbs Sampler
MMX: Mosquito Magnet-X trap
MTR: M-Trap
MTRC: M-Trap + CDC Light Trap
PCR: Polymerase Chain Reaction
RMSE: Root Mean Square Error
SUN: Suna Trap
