# Supplementary material for: A statistical calibration tool for methods used to sample outdoor-biting mosquitoes
Source: Parasit Vectors. 2022 Aug 17;15:293. doi: 10.1186/s13071-022-05403-7 (PMC9386948; doi:10.1186/s13071-022-05403-7)
Supplement: Supplementary file 1 — Additional file 1: Table S1. Summary of all other mosquitoes collected for each trap type. [file 13071_2022_5403_MOESM1_ESM.pdf]

Table S1: Summary of all other mosquitoes collected for each trap type including the Human Landing Catches (HLC)

**Species**	**HLC**	**SUN**	**BGS**	**ITT-C**	**MMX**	**MTRC**	**MTR**
Anopheles arabiensis	5282	1187	531	615	370	2125	1454
Anopheles funestus	226	66	40	76	37	115	115
Anopheles coustani	670	1216	207	3	444	564	314
Anopheles pharoensis	101	906	82	0	69	103	76
Anopheles squamosus	56	196	49	1	99	145	127
Anopheles wellcomi	16	3	3	0	9	72	25
Anopheles ziemani	204	1368	183	0	269	340	156
Culex sp.	7191	3666	3709	4970	1018	7710	6645
Mansonia sp.	2101	4527	1001	111	1961	2314	1273
Aedes aegypti	20	2	5	2	30	7	5
Coquellitidia	240	84	117	14	56	127	229
